# Photographic but not line-drawn faces show early perceptual neural sensitivity to eye gaze direction

**DOI:** 10.3389/fnhum.2015.00185

**Published:** 2015-04-10

**Authors:** Alejandra Rossi, Francisco J. Parada, Marianne Latinus, Aina Puce

**Affiliations:** ^1^Cognitive Science Program, Indiana UniversityBloomington, IN, USA; ^2^Program in Neuroscience, Indiana UniversityBloomington, IN, USA; ^3^Department of Psychological and Brain Sciences, Indiana UniversityBloomington, IN, USA; ^4^Department of Psychiatry, Harvard Medical SchoolBoston, MA, USA; ^5^Institut de Neurosciences de la Timone, UMR7289, CNRS, Aix-Marseille UniversitéMarseille, France

**Keywords:** N170 ERP, real faces, line-drawn faces, gaze aversion, apparent motion

## Abstract

Our brains readily decode facial movements and changes in social attention, reflected in earlier and larger N170 event-related potentials (ERPs) to viewing gaze aversions vs. direct gaze in real faces (Puce et al., 2000). In contrast, gaze aversions in line-drawn faces do not produce these N170 differences (Rossi et al., 2014), suggesting that physical stimulus properties or experimental context may drive these effects. Here we investigated the role of stimulus-induced context on neurophysiological responses to dynamic gaze. Sixteen healthy adults viewed line-drawn and real faces, with dynamic eye aversion and direct gaze transitions, and control stimuli (scrambled arrays and checkerboards) while continuous electroencephalographic (EEG) activity was recorded. EEG data from 2 temporo-occipital clusters of 9 electrodes in each hemisphere where N170 activity is known to be maximal were selected for analysis. N170 peak amplitude and latency, and temporal dynamics from Event-Related Spectral Perturbations (ERSPs) were measured in 16 healthy subjects. Real faces generated larger N170s for averted vs. direct gaze motion, however, N170s to real and direct gaze were as large as those to respective controls. N170 amplitude did not differ across line-drawn gaze changes. Overall, bilateral mean gamma power changes for faces relative to control stimuli occurred between 150–350 ms, potentially reflecting signal detection of facial motion. Our data indicate that experimental context does not drive N170 differences to viewed gaze changes. Low-level stimulus properties, such as the high sclera/iris contrast change in real eyes likely drive the N170 changes to viewed aversive movements.

## Introduction

Successful social behavior requires evaluating incoming sensory information and merging it with situationally relevant behavioral responses. Though a part of our social life may rely on purely reflexive behaviors, specialized neural activity is needed in evaluating social cues (Stanley and Adolphs, 2013). Over the past two decades social neuroscience, the study of social and cognitive influences on biological processes (Cacioppo, 1994; Cacioppo et al., 2000; Ochsner and Lieberman, 2001), has aimed to generate a brain-based understanding of social behaviors. An individual’s social cognitive understanding of the world is likely to not be context-invariant, however, the effects of task and experimental context on social cognition are seldom studied. In the case of social attention, in daily life a gaze change will occur in the context of directed emotions and actions from not only one’s self, but from others around us. This social environment, with many multisensory cues and continually changing context, is difficult to reproduce in a controlled laboratory setting. However, even in a controlled laboratory setting, experimental context can potentially modulate neural responses to particular stimulus conditions or tasks, and may underlie some of the differences observed between studies in the literature. In a laboratory setting, experimental context can be created within a trial, across trials or conditions, or across experimental sessions. Context effects could potentially be driven by the characteristics of the stimuli (bottom-up), or by task demands/instructions to subjects (top-down).

One particularly striking experimental context effect has been reported to viewing faces. It has long been known that the N170 event-related potential (ERP) is strongly driven by the physical or structural characteristics of a face stimulus (Bentin et al., 1996). In an elegant experimental manipulation, Bentin and Golland (2002) recorded an N170 ERP evoked to (static) schematic line-drawings of faces, scrambled versions of the same faces, and line-drawings of common objects. In their design different subject groups were exposed to the stimuli with different block orders. The scrambled, or jumbled, versions of the line-drawn face stimuli had recognizable features, whose position relative to the outline of the face was altered. As expected, N170s were elicited to all stimulus categories, and were significantly larger to the intact schematic faces in both experiments. Critical to the current discussion, significantly larger N170s occurred to jumbled schematic faces but *only* when that stimulus block *directly followed* the schematic face block (Bentin and Golland, 2002), indicating how important stimulus-induced context effects can be in a laboratory setting. In a different study, N170 amplitude elicited to Moonee faces decreased by priming with photographic images of the same individuals represented in the Moonee faces (Jemel et al., 2003). The strongest priming effect occurred to images that were the actual photographic image of the Moonee face stimulus (a bottom-up effect), however, priming was also observed to different real images of the same individual relative to the Moonee faces (top-down effect) (Jemel et al., 2003). As a third example of the importance of experimental context effects, differences in the lateralization of N170 to faces can occur as a function of stimulus conditions used in the experiment. For example, the classic right lateralization of N170 is seen when faces are randomly presented among other object classes (e.g., Bötzel et al., 1995; Bentin et al., 1996; Eimer, 1998; Itier and Taylor, 2004) compared to a bilateral or even left-lateralization pattern when faces are presented in series with other faces (Deffke et al., 2007). These findings caution how important experimental context can be on N170s elicited to faces (Maurer et al., 2008). Indeed, N170 is larger to ambiguous face-like stimuli that are *perceived as faces* relative to the same stimuli when they are not seen as faces (George et al., 1996; Sagiv and Bentin, 2001; Bentin and Golland, 2002; Latinus and Taylor, 2005, 2006). These effects have been proposed to be driven by stimulus context by a number of investigators (Bentin and Golland, 2002; Latinus and Taylor, 2006).

Isolated eyes evoke larger and delayed N170s relative to full faces (Bentin et al., 1996; Jemel et al., 1999; Puce and Perrett, 2003). Hence, the context of the face itself (e.g., outline and other face parts) may affect the neural response elicited to the eye stimulus—an effect that does not occur to presenting other face parts in isolation. Due to its sensitivity to dynamic gaze transitions (Puce et al., 2000; Conty et al., 2007), N170 has been posited to be a neural marker of communicative intent (Puce, 2013). Relevant for the present study, N170s to dynamic *gaze aversions* are larger and earlier than those to gaze transitions looking directly at the observer (Puce et al., 2000; Watanabe et al., 2002; but see Conty et al., 2007). This effect occurs to full images of faces, and isolated eyes (Puce et al., 2000), suggesting that N170 signals changes in social attention, and reflects the potential salience of gaze direction (Puce and Perrett, 2003; Conty et al., 2007).

N170 modulation to dynamic facial movements is not exclusive to eyes: larger N170s occur to mouth opening vs. closing movements—potentially reflecting a response to a pending utterance (Puce et al., 2000), and this effect occurs in both real and line-drawn faces (Puce et al., 2003; Rossi et al., 2014). Unlike in dynamic mouth motion, N170s to gaze aversions are strongly modulated by stimulus type: real faces show N170 differences to averted vs. direct gaze (Puce et al., 2000), whereas line-drawn faces do not (Rossi et al., 2014). These differences beg the question about effects of stimulus-driven context effects on the N170 elicited to dynamic facial movements. Hence, here we recorded N170 ERPs to dynamic gaze transitions to both real and line-drawn dynamic face images, and scrambled controls within the same experiment (using an experimental structure similar to that of Puce et al., 2003). We performed a standard ERP peak analysis, focusing on N170, and reasoned that if stimulus-context effects were driving N170 modulation, we would expect to observe larger N170s to gaze aversion vs. direct gaze for *both* real and line-drawn faces. In contrast, if the N170 effect was driven by low-level stimulus features only in the eye stimuli e.g., high iris/sclera contrast in the real images of faces, then the N170 effect would be seen *only* to dynamic images *using real faces* and not to line-drawn face images. This N170 modulation would not be predicted to occur for real control stimuli, in line with our previous studies. Finally, if the N170 effect was driven by a general low-level effect of local stimulus contrast change (occurring in both face and control stimuli), then we might expect to observe larger N170s to the real faces and their respective controls, relative to the line-drawn stimuli.

As well as examining averaged ERP activity, we also investigated oscillatory electroencephalographic (EEG) behavior post-motion onset to all stimulus types at electrode sited generating maximal N170 activity in a frequency range of 5–50 Hz. Previous studies evaluating facial motion effects have focused exclusively on averaged ERPs, which represent linearly summed EEG trials that are phase-locked to a relevant event (e.g., motion onset), and that are independent of ongoing EEG activity (Jervis et al., 1983). It has been proposed that the transient phase-resetting of ongoing oscillatory EEG activity underlies ERP generation (Brandt and Jansen, 1991). However, oscillatory EEG activity that is *not phase-locked* can also occur to a stimulus event, and will not be seen in an averaged ERP (Makeig et al., 2004). Oscillatory EEG activity expressed both as a function of EEG frequency *and* time relative to stimulus onset and/or execution of motor response can be identified using time-frequency decomposition of EEG signals, and displayed as Event-Related Spectral Perturbation (ERSP) plots (Makeig et al., 2004). Changes in a given EEG frequency band can occur from more than one process or underlying mechanism (e.g., see Sedley and Cunningham, 2013). Modulation of alpha band (8–12 Hz) power has been linked to changes in attentional state (Worden et al., 2000; Sauseng et al., 2005; Thut et al., 2006; Fries et al., 2008), and performance on visual perception tasks (Ergenoglu et al., 2004; Babiloni et al., 2006; Thut et al., 2006). Alpha may act as an inhibitory brain signal (Klimesch, 2012), which might enable timing of processing, and gated access to knowledge, and orientation in time, place, and context (Basar et al., 1997; Palva and Palva, 2007; Klimesch, 2012). Increases in beta band power (12–30 Hz) may reflect maintenance of current behaviorally relevant sensorimotor or cognitive states (Engel and Fries, 2010), whereas gamma band power (>30 Hz) increases may facilitate cortical processing, cognitive control and perceptual awareness (Ray and Cole, 1985; Tallon-Baudry and Bertrand, 1999; Grossmann et al., 2007; Engel and Fries, 2010; but see Sedley and Cunningham, 2013).

Ideally, ERSP and ERP analyses performed *in parallel* could more completely characterize neural activity to different task demands and conditions. However, the relationship between oscillatory EEG activity and ERP activity complex (see Rossi et al., 2014) and is not typically studied. Our previous comparisons of ERP and ERSP activity to viewed dynamic eye and mouth movements in dynamic line-drawn faces showed statistically significant differences for apparent motion in the beta and gamma bands between facial motion conditions, which differed timing and frequency content relative to control motion stimuli (Rossi et al., 2014). Given our previous study, here, we expected to observe oscillatory EEG changes in beta and gamma bands that would occur at different post-motion onset times for facial and control motion stimuli.

## Materials and Methods

### Participants

Seventeen healthy participants provided written informed consent to participate in the study. All participants had normal or corrected-to-normal vision, and were free of a history of neuropsychiatric disorders. The study protocol was approved by the Institutional Review Board at Indiana University, Bloomington (IRB 1202007935).

High-density (256 channel) EEG and behavioral data were collected from all participants, and data from 1 individual had to be excluded from further analysis due to a large amount of artifactual EEG contamination from facial/neck muscle activity, as well as line noise. Hence, data from 16 participants (7 males, 9 females) with an average age of 26 years (range 21–34 years) were submitted for analysis. The 16 participants were right-handed, as assessed by the Edinburgh Handedness Inventory (mean: R64.6, SD: 19) (Oldfield, 1971).

### Stimuli

**Figure 1:**
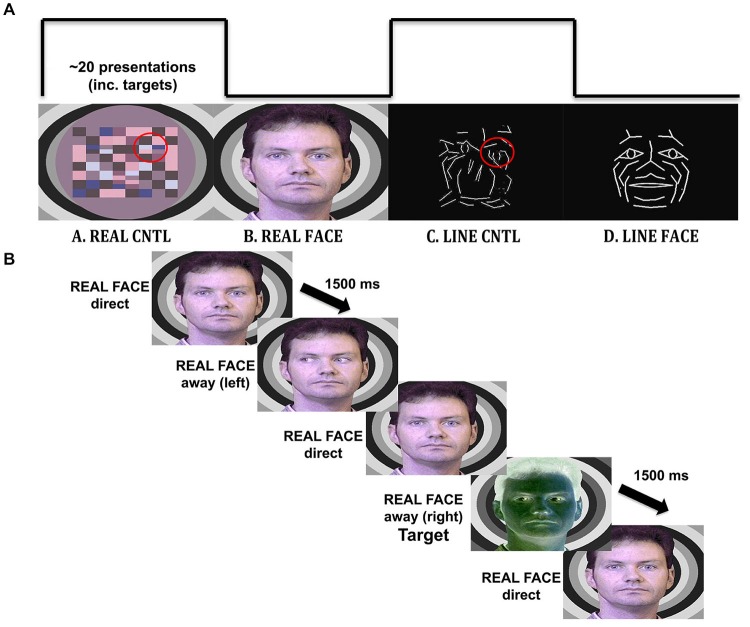
**Materials and methods. (A)** Illustration of the visual stimulation sequence, with example stimuli. Each viewing condition had a 20 s duration and consisted of the alternation of the three images with different gaze direction: direct, away to the left and away to the right. The REAL CNTL condition consists of apparent motion of a checkerboard pattern to simulate eyes moving toward or away from the participant depicted in the REAL FACE condition. A similar correspondence exists (highlighted on red circles for display purposes only) for the LINE CNTL and LINE FACE conditions, where line-drawn images are alternated to display apparent motion in a control and face stimulus. **(B)** Timeline for an experimental trial. An example of a trial sequence in the REAL FACE stimulus block. Participants pressed a mouse button whenever they saw a target (a negative image of the stimulus).

Participants viewed four different types of visual displays alternating between natural images of facial motion and respective motion controls, as well as motion of line-drawn faces and their respective motion controls. The real face, with eyes averting and looking directly at the observer, had a respective motion control that consisted of a colored checkerboard with checks moving towards the left and right in the same visuospatial position as the eyes in the real face (similar to that used in Puce et al., 1998). A line-drawn face, with eyes averting and looking at the observer, had a respective motion control in which line segments in the scrambled stimulus moved with a similar spatial excursion to the eyes, with the same number of pixels contributing to the motion (Figure 1).

#### Stimulus Creation

Real faces had been originally created from still 8-bit color photographs of posed direct and extreme averted (30 degree) gaze positions in both left and right directions. The stimulus face was superimposed on a background of concentric grayscale circles of different luminance. The images were originally created to be presented sequentially to depict dynamic gaze transitions, and mouth motion (see Puce et al., 1998). The corresponding non-facial motion controls consisted of a colored checkerboard pattern that was constructed from hues taken from the original head. Separate corresponding control stimuli were created to that sequential presentation resulted in checks alternating their position in the same regions of the visual field as the eyes in the real face (Puce et al., 1998), and to ensure that subjects did not visualize a “face” in the dynamic control stimulus.

White line-drawn faces on a black background had been originally created from a multimarker recording of facial expressions using specialized biological motion creation software from which lines were generated between some of the point lights [Elite Motion Analysis System (BTS, Milan, Italy)]. The black and white control stimuli had originally been created by extracting line segments from the line-drawn face and spatially re-arranging them in the visual space in an earlier version of Photoshop (Adobe Systems, Inc.), so that the face was no longer recognizable (Puce et al., 2003). The existing line-drawn faces were modified for Rossi et al. (2014) in Photoshop CS5 (Adobe Systems, Inc.) by adding a schematic iris to the face which when spatially displaced could signal a gaze change on the stimulus face. A direct gaze consisted of a diamond-shaped schematic pupil positioned in the center of each schematic eye. Averted gaze consisted of an arrow-shaped schematic pupil that was moved to the extremity of the schematic eye (Figure 1). Thus, by toggling the two schematic eye conditions, observers reported seeing a convincing “direct” vs. “averted” gaze transition in the line-drawn face. Similarly, line-drawn control stimuli were created, using a rearranged “scramble” of the lines making up the eye movements on the face stimuli, ensuring that all stimuli presented would be equiluminant, and have similar motion excursions, as well as contrast and spatial frequency characteristics (Figure 1A). On debriefing post-experiment, subjects did not report seeing “eye” stimuli in the line-drawn control stimulus.

For all stimulus types, the effect of smooth movement was generated and no side-switch transition was possible (e.g., eyes looking to the right followed by eyes looking to the left). Negative-contrast versions (inverse colors) of all the stimulus versions were constructed to be used as infrequently presented targets (Figure 1B).

### Procedure

Participants viewed the stimuli displayed on a 24-inch monitor (Dell Ultra Sharp U2412M, refresh rate of 60 Hz) resulting in an overall visual angle of 5 × 3 (vertical × horizontal) degrees. Participants completed four experimental runs in total; each run lasted approximately 6 min to allow participants to remain still for the EEG recording and maintain their level of alertness. After each run, participants had a self-paced break.

All stimulus types were always presented in each experimental run, with a run consisting of the repeated presentation of the following alternating 20 s stimulus blocks (Figure 1A; following the procedure used in Puce et al., 2003):

1. REAL FACE. Three versions of male face with eyes directly looking at the observer, eyes averted to the left, or eyes averted to the right (Figure 1B) were presented in alternation to produce apparent motion depicting change in gaze position from a direct gaze to an averted gaze position to the left or to the right [gaze aversions: eyes-away] and from an averted gaze back to a direct gaze position [eyes-direct] similar to that used in Puce et al. (1998).
2. REAL CONTROL. Checkerboard patterns were alternated to produce an apparent motion stimulus varying in the same part of the visual field as the eyes in the REAL FACE similar to that used in Puce et al. (1998).
3. LINE FACE. Three versions of line-drawn face stimuli were alternated to change gaze position to look directly at participants [eyes-direct] or avert gaze either to the left or right [eyes-away] as for the REAL FACE condition, similar to that used in Rossi et al. (2014).
4. LINE CONTROL. The spatially “scrambled” versions of the LINE FACE were alternated to produce apparent motion in the same part of the visual field as the eyes in the LINE FACE condition, similar to that used in Rossi et al. (2014).

Stimulus onset asynchrony was randomly varied between 1000 and 1500 ms on each experimental trial (i.e., between two consecutive apparent motion onsets). A total of 210 trials were acquired per stimulus type (LINE FACE, LINE CONTROL, REAL FACE, REAL CONTROL).

The experiment was run using Presentation Version 14 (NeuroBehavioral Systems, 2010). Participant reaction times and accuracy were logged, and time stamps for different stimulus types (as well as button press responses for detected target stimuli) for each trial were automatically sent to the EEG system and stored in the EEG file.

Participants were instructed to press a button indicating the presence of a target stimulus. Target stimuli were negative-contrast versions of all stimuli used in the experiment (Figure 1B). Targets were randomly assigned to each alternating block (20% of trials). Trials with targets were not included in ERP/EEG analyses. Similarly, so as to remove potential confounds created by changes in stimulus type (i.e., for the first stimulus of each block, as well as for stimuli immediately following targets), trials following a target and the first stimuli of each block were not included in ERP/EEG analyses. The purpose of the target detection task was to keep participants attentive. All participants completed a short practice run (36 trials) at the beginning of the session and were given feedback regarding detection of target stimuli. All participants completed the practice run with 100% accuracy. EEG trials from the practice run were not included in subsequent analyses.

### EEG Data Acquisition and Preprocessing

#### EEG Data Acquisition

A Net Amps 300 high-impedance EEG amplifier and NetStation software (V4.4) were used to record EEG from a 256-electrode HydroCel Geodesic Sensor Net (Electrical Geodesics Inc.) while the participant sat in a comfortable chair and performed the task in a dimly lit, humidified room. Continuous 256-channel EEG data were recorded with respect to a vertex reference using a sampling rate of 500 Hz and bandpass filter of 0.1–200 Hz (the ground electrode was sited on the midline parietal scalp). Stimulus delivery and subject behavioral responses were time-stamped onto all EEG files. Impedances were maintained below 60 kΩ as per the manufacturer’s recommended guidelines. Impedances were tested at the beginning of the experimental session and then once more at the half-way point of the experiment, allowing any high-impedance electrode contacts to be adjusted if necessary.

#### EEG Data Preprocessing

EEG data were first exported from EGI Net Station software as simple binary files. The same pre-processing procedure was applied to the ERP and ERSP analyses. All EEG pre-processing procedures were performed using functions from the EEGLAB toolbox (Delorme and Makeig, 2004) running under MATLAB R2010b (The Mathworks, Natick, MA). EEG data were first segmented into 1700 ms epochs: 572 ms pre-stimulus baseline and 1082 ms after apparent motion onset. EEG amplitude at each trial was normalized relative to the pre-stimulus baseline based on the event-markers, identifying each trial type. ERP data were displayed using a 200 ms pre-motion onset and 600 ms after the motion transition—see Figure 3. [A manufacturer-specified latency correction factor was applied to all behavioral data and epoched ERP data. In our case, given a sampling rate of 500 Hz, a correction of 18 ms was made, as per manufacturer guidelines].

EEG epochs were first visually inspected to identify and exclude bad channels from each individual subject EEG dataset. The electrodes identified as bad differed between subjects; average number of “bad” electrodes was 22 ± 2.15 (standard error of mean) out of 256 channels. Epochs with very large artifacts (e.g., very large subject movements and channel drifts) were manually rejected prior to subjecting the EEG data subsequent artifact detection analyses.

Independent Component Analysis (ICA) was used to identify and subtract components representing artifacts such as eye movements, eye blinks, carotid pulse, muscle activity and line-noise (Bell and Sejnowski, 1995; Delorme and Makeig, 2004). This allowed trials with eyeblinks to be adequately corrected, and allowed these trials to be included in the analysis. A total of 32 ICA components were generated for each participant’s EEG dataset. Eyeblinks, cardiac artifact and muscle activity were identified in isolated ICA components. Following removal of artifactual ICA components and reconstitution of the EEG signal, interpolation of bad channels was performed to regenerate a 256-channel EEG dataset. Bad channels were interpolated using a spherical interpolation: electrical activity was interpolated with respect to the surrounding nearest neighbor electrodes.

Data were re-referenced to a common average reference. ERP components such as the N170 and the vertex positive potential (VPP) amplitude have previously been shown to be very sensitive to reference location (Joyce and Rossion, 2005). The average reference has been suggested as being optimal as it captures finer hemispheric differences and shows the most symmetry between positive and negative ERP peaks for face-related stimuli (Joyce and Rossion, 2005). Only behaviorally correct EEG trials, i.e., no false alarms for targets, were included in subsequent analyses.

### EEG/ERP Data Analyses

**Figure 2:**
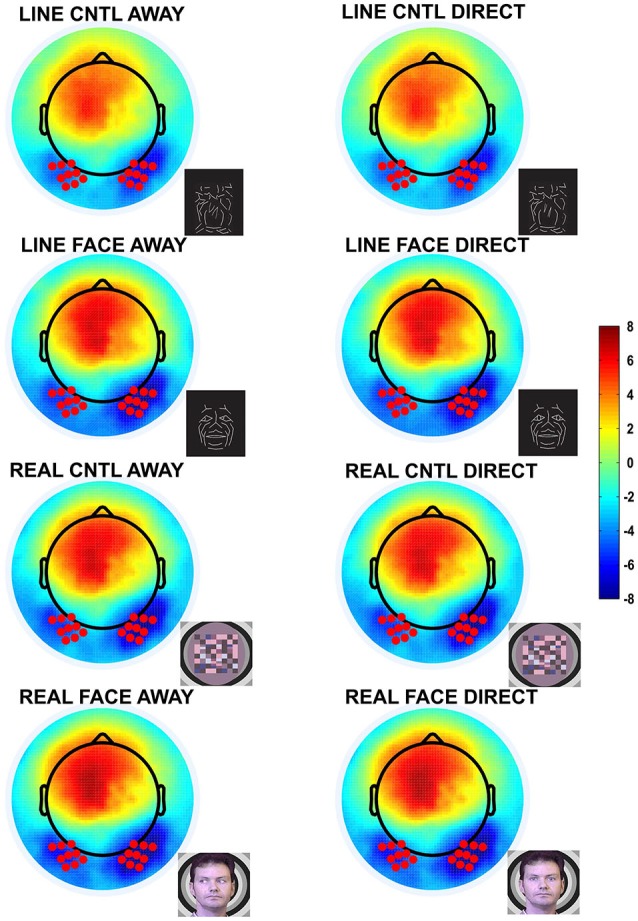
**Group data: Topographic voltage maps of peak N170 activity**. The N170 is distributed across the bilateral occipitotemporal scalp and appears in all conditions, including the control conditions. The topographic maps are displayed in a top-down view with nose at top and left hemisphere on the left. Color scale calibration bars depict amplitude in microvolts. Red circles on the maps depict the 9 electrodes in each hemispheric cluster that provided input for N170 statistical analyses. Small black dots depict additional sensor locations.

**Figure 3:**
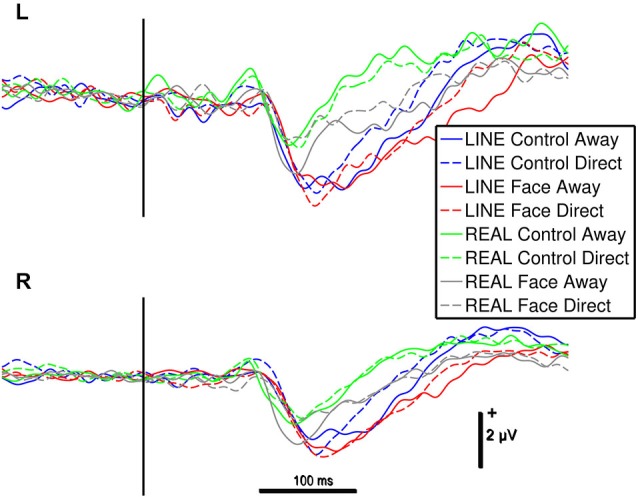
**Group data: ERPs from left (L) and right (R) occipitotemporal electrode clusters as a function of stimulus type**. An epoch of activity is shown from 200 ms pre-stimulus onset to 600 ms post-stimulus. Legend: Line colors indicate corresponding stimulus type shown LINE CONTROLS in blue, LINE FACES in red, REAL CONTROLS in green and REAL FACES in gray. The vertical black bar superimposed on the ERP waveforms denotes motion onset. Vertical and horizontal calibration bars denote amplitude in microvolts and time in milliseconds, respectively.

Two temporo-occipital 9 electrode clusters including equivalent 10–10 system sites P07/P9 and P08/P10 were chosen for further analyses, based on inspection of the grand averaged data from the current study and previously reported maxima in N170 amplitudes that used 4 electrode clusters for 64- and 128-channel EEG derivations, and P09 and P10 for smaller electrode arrays of 10–10 system sites (Puce et al., 2000, 2003; Carrick et al., 2007; Brefczynski-Lewis et al., 2011; Rossi et al., 2014; Figures 2, 3). Averaged data from the 9 electrodes in each hemispheric cluster were used in all subsequent ERP analyses. Similarly, single-trial EEG data recorded from the same 9 electrodes in each hemispheric cluster were used for ERSP analysis.

#### Analysis of Event-Related Potentials

A digital 40 Hz infinite impulse response (IIR) low-pass filter was applied to the artifact-free behaviorally correct EEG data. Average ERPs were generated for each of the eight conditions and for each subject (about 200 trials per condition per subject on average). The ERPs from all subjects were averaged to generate a grand-average set of ERP waveforms for each condition and EEG channel.

Data from each 9 electrode temporo-occipital cluster were extracted and the average time-course for each electrode cluster was generated for subject and condition, and was subsequently used for calculating N170 amplitude and latency. In line with previous work (Puce et al., 2003), we focused on the N170 as a neural marker of the perception of facial motion. In our data, consistent with previous studies, the N170 showed a lateralized posterior scalp distribution (Figure 2). Using an automated peak detection procedure within a search time window of 150–250 ms after apparent motion onset, N170 peak amplitudes and latencies were extracted for each condition, each subject, and each electrode cluster independently.

#### Analysis of Event-Related Spectral Perturbations

All analyses were performed using custom in-house routines written using the EEGLAB toolbox (Delorme and Makeig, 2004) running under MATLAB. Artifact-free, behaviorally correct EEG segments were convolved with a linearly increased Morlet-wavelet on a trial-by-trial basis for each condition and subject. Specifically, the length of the wavelet increased linearly from 1 to 12 cycles across the frequency range of 5–50 Hz (theta, alpha, beta, and low-gamma). The linear increment of wavelet cycles is a commonly used practice when calculating spectral components in neurophysiological data, so that temporal resolution can be comparable for lower and higher EEG frequencies (Le Van Quyen et al., 2001) (for a detailed account on spectral analyses of EEG see Herrmann et al., 2005). After the EEG signals in each trial were convolved with a Morlet wavelet, they were transformed into power, and the resulting values were then averaged across trials. We analyzed the spectral power of components in the theta (5–8 Hz), alpha (8–12 Hz), beta (12–30 Hz), and low-gamma (30–50 Hz) EEG frequency-bands as they evolved over the post-movement epoch. In order to extract even-related spectral power from raw power, a standard baseline procedure was applied in a trial by trail basis (Grandchamp and Delorme, 2011). The window used as baseline comprised data points between −200 and 0 ms pre-stimulus range.

Induced activity is defined as EEG activity that is elicited to the stimulus, but may not be precisely time- or phase-locked to the stimulus transition (in this case apparent motion onset). However, each individual EEG epoch will also contain evoked activity, hence a calculation of “total power” (i.e., sum of evoked and induced activity) in each frequency band was made for EEG epochs in our study (see Tallon-Baudry et al., 1996).

As we have previously noted differences between facial motion stimulus type for eye and mouth movements (Rossi et al., 2014), we performed a similar analysis and generated differential ERSP plots between pairs of conditions: LINE CONTROL Direct vs. Away, LINE FACE Direct vs. Away, REAL CONTROL Direct vs. Away and REAL FACE Direct vs. Away.

#### Statistical Testing for Significant Differences

##### ERP peak analysis

Differences in temporo-occipital N170 peak amplitude and latency were evaluated using a 4-way repeated-measures ANOVA with Hemisphere (Left, Right), Configuration (Face, Control), Stimulus Type (Real, Line) and Motion (Away, Toward) as within-subjects factors using SPSS for MAC 18.0 (SPSS Inc.). Significant main effects were identified at *P* values of less than 0.05 (after Greenhouse-Geisser correction). Contrasts were evaluated using the Bonferroni criterion to correct for multiple comparisons with *P* values of less than 0.05 identifying significant effects.

Furthermore, to specifically assess the specificity of the effect to REAL FACE stimuli, we performed paired *t*-tests between each motion conditions for each stimulus condition. Four *t*-tests were performed (i.e., REAL FACE AWAY × REAL FACE DIRECT, REAL CONTROL AWAY × REAL CONTROL DIRECT, LINE FACE AWAY × LINE FACE AWAY, LINE CONTROL AWAY × LINE CONTROL DIRECT). The level of statistical significance (*a priori* two-tailed) was set at *p* < 0.05.

##### ERSP analysis

To measure the complete temporal extent of effects over frequency, we used a bootstrap approach (*N* = 1000 bootstraps), identifying time-frequency data points of statistically significant differences based on data-driven 95% confidence intervals (as described and implemented in Pernet et al., 2011) from the data of the two 9 electrode clusters. Non-parametric permutation was used to estimate the distribution under the null hypothesis of no differences in oscillatory amplitude between the pair of conditions.

Due to our paired design, when a subject was selected randomly, results from all his or her conditions were included in that sample. For each condition, we averaged the data across (resampled) participants and computed differences between conditions. Thus, for each one of the observed mean differences between conditions for a given frequency at each time-point, a *t*-statistic was calculated. At this stage, time points were evaluated according to a threshold set if their t-statistic corresponded to a value below 0.05 according to the Student’s t-distribution. This procedure was repeated for all ERSP time-points at each frequency. Temporally contiguous threshold time points were grouped into temporal clusters. At each bootstrap iteration, the temporal cluster mass was computed as the sum of the *t*-statistics over consecutively significant time-points, with the maximum cluster mass being recorded. Finally, temporal clusters in the observed data were deemed significant if their mass exceeded the maximum cluster mass of 95% of all bootstrap replicates (corresponding to a significance level of 0.05). This method allowed correction for multiple comparisons. Thus, the cluster mass statistic identified temporal regions with significant differences while avoiding false-positives arising from multiple comparisons (Pernet et al., 2011). This approach is comparable to false-discovery rate (FDR; Benjamini and Hochberg, 1995).

## Results

### Behavioral Data

Participants identified the target stimulus (image negative) with 99% accuracy by button press. Mean reaction time for target detection by stimulus type was 557 ± 94 ms (s.d.) for REAL faces, 577 ± 112 ms for REAL controls, 607 ± 113 ms for LINE faces and 560 ± 92 ms for LINE controls. A 2-way (Configuration × Stimulus Type) repeated-measures ANOVA showed a significant main effect of Stimulus Type (*F*_(1,15)_ = 11.067, *P* < 0.001) and an interaction effect of Configuration by Stimulus (*F*_(1,15)_ = 26.24, *P* < 0.001).

For the significant main effect of Stimulus Type, REAL stimuli (real faces and real controls) generated faster responses relative to LINE stimuli (line-drawn faces and line-drawn scrambled controls, mean difference: 30 ± 9 ms). The significant interaction effect of Configuration by Stimulus type indicated that real faces generated a faster response compared to REAL controls (mean difference: 47 ± 11 ms), while the opposite was seen for line-drawn stimuli, line-drawn controls were identified fastest (mean difference: 47 ± 13).

The current behavioral task was used to help participants pay attention to the display. EEG epochs from these target trials were not included in subsequent analyses.

### Peak Analysis of the N170 ERP

N170 amplitudes and latencies were extracted from each of the two temporo-occipital scalp electrode clusters for each participant and condition for subsequent statistical testing. N170 was maximal over the temporo-occipital scalp, as demonstrated by the topographic voltage maps (Figure 2) plotted at the time point at which the N170 was maximal in amplitude. N170 was elicited in all stimulus conditions (Figure 3).

N170 latency and amplitude data for each condition and hemisphere are shown in Table 1. A 4-way repeated-measures ANOVA for N170 peak amplitude differences revealed a significant main effect for hemisphere (*F*_(1,15)_ = 11.265, *P* < 0.001) and stimulus type (Real vs. Line; *F*_(1,15)_ = 46.289, *P* < 0.001). The main effects for configuration (Face, Control) and motion (Away, Direct) were not significant. A significant interaction effect was observed between stimulus type and motion (*F*_(1,15)_ = 5.143, *P* < 0.05).

**Table 1:** **Group N170 peak amplitude (μV) and latency (ms) data: Mean and Standard Errors (Std) as a function of hemisphere (Hem) and Condition**.

Hem	Condition	Peak Ampl (μV)	Std	Peak Lat (ms)	Std
**Left**	REAL FACE away	−1.45	0.24	209.68	10.97
	REAL FACE direct	−1.13	0.15	216.75	8.97
	REAL CTRL away	−0.12	0.26	207.25	10.12
	REAL CTRL direct	−1.14	0.20	200.87	8.91
	LINE FACE away	−1.66	0.25	236.06	8.89
	LINE FACE direct	−1.73	0.30	232.81	6.35
	LINE CTRL away	−1.63	0.16	234.37	9.65
	LINE CTRL direct	−1.87	0.24	233.18	7.28
**Right**	REAL FACE away	−2.24	0.25	218.12	7.72
	REAL FACE direct	−1.64	0.20	223.18	8.96
	REAL CTRL away	−1.86	0.28	203.06	7.52
	REAL CTRL direct	−1.69	0.26	200.81	7.25
	LINE FACE away	−2.54	0.34	246.81	7.37
	LINE FACE direct	−2.64	0.27	236.50	6.97
	LINE CTRL away	−2.18	0.32	236.62	8.44
	LINE CTRL direct	−2.42	0.36	231.87	5.87

*Legend: Ampl = amplitude; Lat = latency*.

For the significant main effect of hemisphere, *post hoc* paired comparisons revealed that N170 amplitude was greater for the Right hemisphere relative to the Left Hemisphere (mean difference: 0.66 ± 0.26 μV). The main effect of stimulus type showed that N170 amplitude was greater for the line-drawn stimuli relative to the real stimuli (mean difference: 0.55 ± 0.08 μV, Figure 3). *Post hoc* comparisons for the interaction effect between stimulus type and motion revealed that among the REAL stimuli (i.e., real faces and real controls) the N170 for averted gaze was significantly larger than that to direct gaze (mean difference: 0.29 ± 0.13 μV) (Figure 3); an effect not seen for line-drawn stimuli.

One could argue that our 4-way ANOVA would reveal a 3-way interaction between stimulus type, configuration, and motion. This was not the case. When we compare our current data to those of Puce et al. (2000); we note that the authors also did not find interaction effects on N170 amplitude as assessed by means of 3-way ANOVAs performed at isolated hemispherically homologous electrode sites in a study that was performed using only 22 EEG electrodes. To try and investigate potential differences and similarities between the two studies, we further explored our current high-density EEG data by running paired *t*-tests on N170 amplitude for the Away and Direct motion transition for each stimulus type and configuration in the right occipitotemporal cluster (given that the main differences in the original study were reported in the right hemisphere). These analyses indicated that N170 amplitude was significantly larger for Away relative to Direct for REAL FACES (*t*_(15)_ = −2.229, *P* = 0.04, mean difference = 0.46 μV), consistent with the difference reported in Puce et al., 2000. In contrast, N170 amplitudes for LINE faces were not significantly different [LINE FACE Away relative to LINE FACE Direct (*t*_(0.15)_ = 0.411, *P* = 0.69, mean difference = 0.08 μV)]. Similar comparisons across the control conditions were also not significant: REAL controls showed no differences between conditions [REAL CONTROL Away relative to Direct (*t*_(15)_ = −0.85, *P* = 0.41, mean difference = −0.11 μV] and LINE controls also showed no significant differences in N170 amplitudes [LINE CONTROL Away relative to Direct (*t*_(15)_ = 0.241, *P* = 0.12, mean difference = 1.6 μV].

The 4-way repeated-measures ANOVA for N170 latency revealed a significant main effect of Stimulus type (*F*_(1,15)_ = 39.49, *P* < 0.001) and Configuration (*F*_(1,15)_ = 7.773, *P* < 0.05). No other statistically significant main effects of hemisphere or motion, or interaction effects were observed for N170 latency. For the significant main effect of Stimulus type, *post hoc* comparisons indicated that the effect might have been driven by the shorter latencies to REAL faces compared to LINE faces (mean difference: 26 ± 4 ms, Figure 3). For the significant main effect of Configuration, *post hoc* comparisons suggested that N170s for CONTROL (both REAL and LINE) were shorter compared to FACE stimuli (mean difference: 9 ± 3 ms, Figure 3). To statistically evaluate these differences, four paired *t*-tests were performed (see Section Materials and Methods). None of these latency differences were found to be statistically significant [REAL FACE Away relative to REAL FACE direct (*t*_(15)_ = 0.979, *P* = 0.343, mean difference = 6.06 ms; LINE FACE Away relative to LINE FACE direct (*t*_(15)_ = 1.563, *P* = 0.139, mean difference = 6.78 ms; REAL CONTROL away relative to REAL CONTROL direct (*t*_(15)_ = 1.140, *P* = 0.272, mean difference 4.31 ms; LINE CONTROL away relative to LINE CONTROL direct (*t*_(15)_ = 0.457, *P* = 0.654, mean difference = 2.96 ms)].

### Temporal Dynamics: ERSP Plots

**Figure 4:**
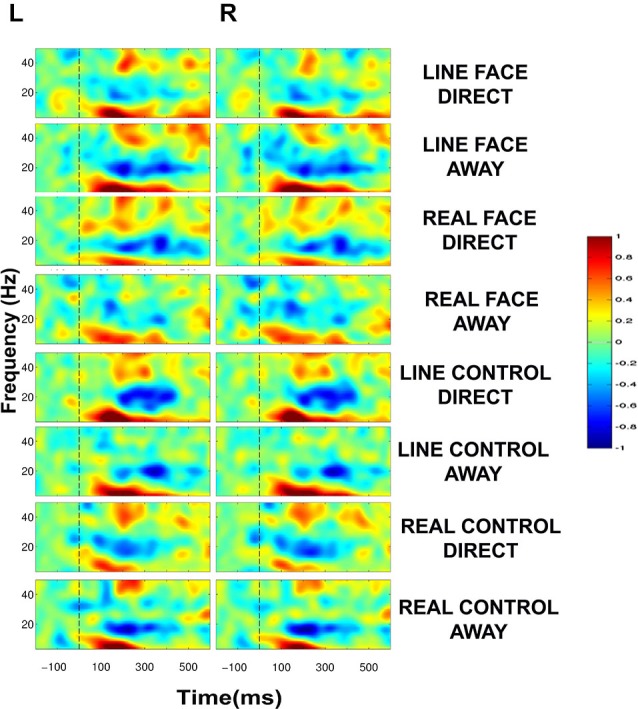
**Group data: Event-Related Spectral Perturbation (ERSP) plots as a function of condition and hemisphere**. Left (L) and right (R) occipitotemporal data are presented in left and right columns, respectively. In each four-part display panel total ERSP activity is shown for each respective stimulus condition. The *y*-axis displays frequency (Hz) and the *x*-axis displays time (ms). Power (decibels) of ERSP activity in decibels, being a default unit used in analysis packages such as EEGLAB (Delorme and Makeig, 2004), is depicted by the color calibration bar at the right of the figure. The vertical broken line at time zero indicates the apparent motion stimulus onset.

ERSP plots demonstrated clear activity in selected EEG bands in all conditions in the post-motion onset period (Figure 4). The activity profile was similar across all conditions, including activity spanning over multiple time points and frequency bands. A common feature across all conditions was a prolonged burst of activity in the theta (5–8 Hz) and alpha (8–12 Hz) frequency bands in both electrode clusters extending from ~100–400 ms. Moreover, a consistent decrease in amplitude in the beta range (12–30 Hz) was also seen for most conditions extending from ~150–400 ms. An additional feature in the ERSP plots was activity in the low-gamma band (30–50 Hz) peaking roughly around 200 ms after apparent motion onset for most conditions (Figure 4).

**Figure 5:**
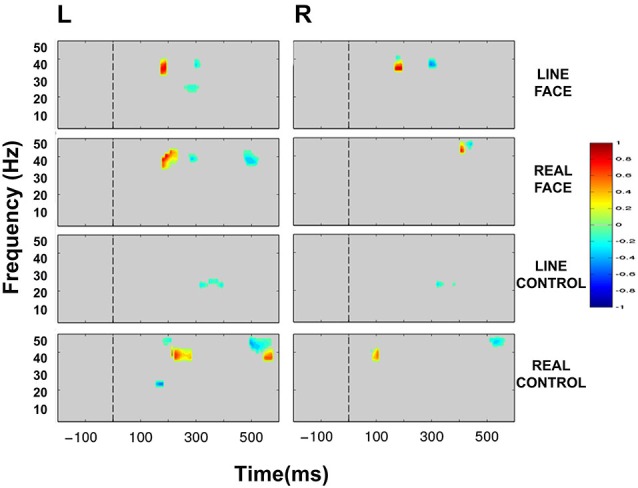
**Group data: Statistically significant ERSP plot differences between stimulus conditions as a function of hemisphere**. Left (L) and right (R) occipitotemporal data are presented in left and right columns, respectively. LINE FACE, REAL FACE, LINE CONTROL and REAL CONTROL difference plots appear from top to bottom panels, respectively. For LINE FACE, LINE FACE Away was subtracted from LINE FACE Direct, and for REAL FACE, REAL FACE Away was subtracted from REAL FACE Direct. For LINE CONTROL, the ERSP plot from LINE CONTROL Away has been subtracted from LINE CONTROL Direct. For REAL CONTROL, REAL CONTROL Away was subtracted from REAL CONTROL Direct. Frequency (Hz) is displayed on the *y*-axis as a function of time (ms). The *direction* of the difference in spectral power is depicted by the color calibration bar at the right of the figure. Warm colors depict increased power for condition 1 (Direct) whilst cool colors indicate increased power for condition 2 (Away). Gray areas in the plot indicate regions where the differences between conditions were not significant. The vertical broken line at time zero indicates the apparent motion stimulus onset.

Statistically significant differences between conditions were seen only in the beta (12–30 Hz) and low-gamma (30–50 Hz) frequency bands. Figure 5 displays masked time-frequency statistically significant differences between facial motion transitions and control motion transitions. We discuss these differences for each stimulus type below.

#### Line Face

ERSP comparisons between gaze transitions for LINE FACE stimuli produced statistically significant differences in beta and gamma bands in the interval of 150–300 ms post-motion onset in both electrode clusters (Figure 5). Larger bilateral gamma amplitudes (~30–~40 Hz) were seen to direct gaze transitions (LINE FACE Direct) peaking at around 200 ms relative to gaze aversions (LINE FACE Away) (Figure 5 first row). For the opposite side of the contrast, larger bilateral gamma activity at ~40 Hz occurred at a later point in time (at ~300 ms for LINE FACE Away vs. Direct, Figure 5 first row). Significant differences in beta activity were only observed in the left temporo-occipital electrode cluster, with a relative larger decrement in beta amplitude for Away relative to Direct.

#### Real Face

REAL FACE stimuli elicited divergent significant differences across hemispheres (as shown in Figure 5 s row) relative to LINE FACES. In the left electrode cluster, statistically significant differences occurred at similar times and were confined to the same frequency range, and were similar for REAL and LINE face stimuli. Direct gaze changes in REAL FACES elicited larger gamma amplitudes peaking at ~200 ms (~30–45 Hz range) relative to gaze aversions, while Away gaze changes elicited stronger gamma power at ~300 ms (~40 Hz), and at ~500 ms (~35–45 Hz range). In the right electrode cluster, a later biphasic difference in gamma amplitude consisted of initial augmentation and then suppression of activity for direct relative to averted gaze. These effects occurred for frequencies ~40 and ~50 Hz and peaked between 400 ms (Figure 5, second row). Unlike in the left hemisphere, effects for REAL FACES occurred at later times relative to LINE FACES: gamma effects for REAL FACES occurred later in time relative to LINE FACES.

#### Line Control

Unlike for the face stimuli significant effects were effectively confined to the beta range, for LINE CONTROL stimuli identified significant bilateral differences that were confined to the beta band, consisting of brief periods between ~300 to ~400 ms after movement onset were observed bilaterally (Figure 5, third row). This difference was driven by stronger beta suppression in the Away condition in both hemispheres.

#### Real Control

REAL CONTROL stimuli generated a much more diverse pattern of differences extending between ~100 to ~600 ms after movement onset in beta and gamma activity in the left hemisphere (Figure 5, fourth row) relative to LINE CONTROL stimuli. First, significantly stronger activity for the REAL CONTROL Away condition consisted of an early beta component peaking right before 200 ms (~20–25 Hz), and a gamma component at ~200 ms (~45–50 Hz). In this same time range, gamma at ~35–40 Hz between ~200–300 ms, was significantly larger for Direct condition. Later in time, gamma burst extending from ~40–50 Hz occurred between ~500–600 ms and was stronger for the Away condition, while a lower frequency gamma component extending between ~35–40 Hz was significantly stronger between ~550–600 ms for the Direct condition (Figure 5, fourth row). Unlike the left electrode cluster, the right cluster showed more limited significant differences in oscillatory activity between conditions. Specifically, a very early gamma band response peaked at ~100 ms, being stronger for the Direct condition, and a later higher frequency gamma component at ~45 Hz peaking at ~500 ms was stronger for the Away condition.

## Discussion

### ERP Data: N170 Effects

Our main purpose for the experiment was to look for stimulus-induced context effects that might produce modulations of the N170 by gaze transition in line-drawn faces, when presented with real images of faces in the same experiment. For a stimulus context effect to be present, we would expect to observe parallel effects in the form of larger N170s to gaze aversions vs. direct gaze for both real and line-drawn faces. Given our experimental design, this would translate to a significant interaction effect of Motion [Away, Direct] × Config [Face, Control]. If, however, N170 modulation occurred only to gaze changes in real faces, then we would expect to see a significant interaction effect of Motion [Away, Direct] × Config [Face, Control] × Stimulus [Real, Line]. Finally, if N170 modulation was driven by general low-level effects of stimulus luminance and contrast, then we might expect to observe a significant main effect of Stimulus type [Real, Line].

Interestingly, our analysis generated effects that were more complex than predicted for N170 amplitude. We observed a significant main effect of Motion [Away, Direct] × Stim [Real, Line]—which was not what we had predicted. The nature of these differences was clarified with paired *t*-tests, which indicated that N170 was larger for averted vs. direct gaze *only* for real faces—consistent with our previous study (Puce et al., 2000). Also consistent with our previous work (Rossi et al., 2014) there was no effect of gaze aversion on N170 in our line-drawn face stimuli (or control stimuli). Having said that, there were other striking differences in the current dataset that resulted from our initial predictions not being upheld. These, results raise a number of interesting questions about the nature of stimuli and experimental designs, which we subsequently discuss in detail.

However, relative to our original experimental question, based on the above findings we would argue that, stimulus-context effects from real faces were not present for viewed eye movements in impoverished faces, when both stimulus categories are presented within the same experiment. This suggests that the difference in N170 amplitude gaze changes in real faces might be driven by a different neural mechanism relative to N170 modulation by mouth movements. We previously reported to mouth opening vs. closing movements in *both* real and line-drawn faces produce N170 amplitude modulations (Puce et al., 2003). Consistent with what we had previously postulated, it appears that information from mouth movements can be accessed from *both* real and impoverished images, unlike information from the eyes that appears to require real faces.

Why would ERPs elicited to impoverished mouth movements behave so differently to those observed to gaze transitions? Bassili (1978, 1979) originally reported behavioral data to viewed emotional expressions on point-light face stimuli. Success in recognizing different emotions was driven by the subject focusing on either the upper or lower regions of the impoverished face (i.e., eyes/brows vs. mouth, respectively) (Bassili, 1979). The line-drawn stimuli in this study can also be regarded as biological motion stimuli (see Oram and Perrett, 1994, 1996). Most typically, these impoverished forms of stimuli are used to represent very effectively the articulated motion of the joints of the body—where information related to the type of activity being observed can be readily identified from seeing these minimalist displays (Blake and Shiffrar, 2007). There is a very large literature demonstrating the sensitivity of the human brain to biological motion stimuli (see reviews by Giese and Poggio, 2003; Puce and Perrett, 2003; Blake and Shiffrar, 2007). Despite this, very few research groups have studied brain responses to biological motion stimuli involving the face (see the original studies of Bassili, 1978, 1979). Similar to movements of the body, mouth movements are a type of articulated motion. Mouth opening and closing occurs due to the actions of the articulated mandible. Hence, we argue that our previously reported ERP data that demonstrate differences between mouth opening and closing are representing a brain response to *articulated biological motion* (e.g., see Beauchamp et al., 2002; Peuskens et al., 2005).

Other facial movements involving the forehead and eyes do not require the movements of articulated joints, and gaze aversions also fall into this category. Changes in the eyes, either associated with gaze aversions, or with emotions such as fear, surprise, and happiness alter the amount of seen eye white area, which can modulate the brain’s response to these types of stimuli even when these observed changes are task-irrelevant (Whalen et al., 2004; Hardee et al., 2008). This is likely to be driven by the high-contrast human iris-sclera complex. A gaze change, such as a lateral gaze shift, produces a local visuospatial luminance/contrast change. This type of stimulus, which can readily be seen at a distance, is thought to have evolved for the purposes of facilitating social interactions (Emery, 2000; Rosati and Hare, 2009). Human eyes are unique among primates with respect to this attribute, with most other species showing very little difference in contrast between irises and sclera (Rosati and Hare, 2009).

The lack of demonstrated N170 differences to gaze aversions relative to direct gaze transitions in this and our previous study using line-drawn faces (Rossi et al., 2014) supports the idea that neural activity to eye gaze transitions in real faces might be triggered by low-level stimulus features. Specifically, changes in local visual contrast and increased eye white area (see also Whalen et al., 2004; Hardee et al., 2008) as irises/pupils move from a direct to an averted position likely drive the N170 differences previously reported by Puce et al. (2000) and also seen to the real faces in the current study. This is likely to be driven by the high-contrast human iris-sclera complex. We found only a 2-way interaction on N170 amplitude suggesting that a contribution of low-level visual features on the modulation of the N170 cannot be ruled out—as indicated by a discernable ERP to the motion control stimuli. The real control stimuli also presented a high local luminance/contrast difference in the same part of the visual space as did the eye stimulus in the real face. However, based on the paired *t*-test data the local high contrast effect cannot totally account for the observed modulation of N170 amplitude to gaze motion: no differences were seen between N170s to the “Away” and “Direct” transitions for real control stimuli. The local high-contrast of the iris/sclera complex probably contributes to the N170, but cannot explain differential effect seen on the N170 to the real face stimuli. It may well be that the actual configuration of the eye plays a role in the response. This, in some ways, could be regarded as a low-level feature also: a feature that is embedded in a more complex stimulus (the dynamic face). A set of studies with parametric manipulations of these variables would be required to get to the bottom of this effect. Additionally, it would be interesting to investigate the relative effect of iris/sclera contrast and the configuration effect on the N170 by using for instance faces where human eyes were replaced by non-human primate eyes (Dupierrix et al., 2014), or examining responses to non-human primate gaze changes, as typically the iris/sclera complex in the eyes of the non-human primate do not show these high local contrast differences (Emery, 2000; Rosati and Hare, 2009).

Viewing the gaze changes of another individual are thought to produce reflexive changes in one’s visuospatial attention (Hietanen et al., 2008; Itier and Batty, 2009). It could be argued that both the eyes and their respective scrambled controls might cue participants’ visual attention in the motion direction. This possibility has been discussed in the literature (Grossmann et al., 2007; Hadjikhani et al., 2008; Straube et al., 2010). Our line-drawn controls in their “averted” state looked like arrows (facing left and then right), and N170 did not differ between the “arrow” control and the direct gaze control condition (a diamond shape). Behavioral and ERP studies of visuospatial cueing paradigms using Posner-like tasks (Posner, 1980) in healthy subjects have demonstrated similar behavioral effects for both arrows and schematic eyes, but different ERP-related effects that most typically occur *beyond* the P100 and N170 that are specific to these visuospatial cueing tasks. Specifically, anterior and posterior negativities have been described to arrow and schematic gaze-cues respectively (Hietanen et al., 2008; Brignani et al., 2009). Interestingly, when real faces are used in a gaze-cueing paradigm, differences in early ERPs, such as P100 and N170 (P1 and N1) have been reported, producing larger amplitudes for valid trials (Schuller and Rossion, 2001). These experimental results, despite being generated in different experimental designs, are consistent with the current study in that schematic eyes do not elicit changes in earlier sensory ERPs such as N170. This finding bears further investigation, given that the schematic eyes in the visuospatial cueing studies did have contrast between “irises” and “sclera”, unlike those in our current study.

A further point needs to be made on the issue of the schematic representation of faces. Our participants all reported that they found the gaze transitions in both types of faces to be compelling. However, some interesting differences in behavior and neurophysiology were observed for the impoverished stimulus categories. Subjects detected target stimuli that consisted of image negatives for all presented stimulus types. Participants were slower at detecting impoverished face targets relative to real face targets, and were the slowest for impoverished faces relative to impoverished controls. For real stimuli, face targets were identified faster relative to controls. We cannot directly relate our behavioral data to our ERP findings: the ERPs were recorded to trials where no behavioral response was recorded, so we can only speculate about the potential nature of our ERP findings to the impoverished stimuli. One consideration might be that the impoverished faces in the current study might not be treated as faces by the brain. A stimulus such as an impoverished face might be ambiguous, and would hence take a longer time to be evaluated and might require more detailed processing. This might manifest as increased response time (for the detection of targets), as well as increased N170 latencies (which were seen as a main effect for line-drawn vs. real stimuli). Coupled with the longer latency is also an increased N170 amplitude (seen as a main effect for stimulus type for line faces and controls). The increased N170 latencies and amplitudes observed here might potentially reflect the more effortful processing that might be required of these stimuli.

Some intriguing differences in N170 activity relating to data of our control stimuli need to be addressed. In our original study, the checkerboard controls had movements that were *not congruous* with one another i.e., checks changed in two locations corresponding to each eye in the real face, but moved in opposite lateral directions (Puce et al., 1998). The control stimuli were created deliberately in this fashion, as in the piloting of data for the earlier study, subjects reported a very convincing and persistent illusion of eyes that the checkerboard control stimuli created. This created the unwanted confound of visualization in the study. This effect was abolished by introducing a movement condition where checks reversed in opposite directions, and we used this control stimulus in our previous study (see Puce et al., 1998). In the current study we were concerned that differences in the *type* of presented motion (congruous vs. incongruous) across the stimulus conditions may have, in part, contributed to the differences in the neurophysiological response between faces and controls. Therefore, we chose to have congruous motion for all stimulus types. Interestingly, in doing so we may well have created a *stimulus-context effect for the control stimuli—*and have potentially allowed subjects to “see” eyes in the control stimuli (as we had previously experienced). This occurred *only* for the REAL CONTROL stimuli, and not for the LINE CONTROL stimuli—there were no differences related to movement direction. So, we might have actually created an unexpected effect of stimulus-context in this experiment, with the REAL FACES providing a context for the REAL CONTROLS (not unlike that seen by Bentin and Golland, 2002). Our original purpose for running the experiment was to explore context effects related to eye gaze changes in LINE FACES in the presence of REAL FACES. In this latter case, we can state that no effect of stimulus context was observed.

The effects that are induced in N170 activity here underscore how important low-level stimulus manipulations related to luminance/contrast and also motion can be, and these have the potential to interact with task-related variables. Hence, control stimuli of multiple types may have to be used in an experiment so as to understand the nature of observed differences in neurophysiological data across different conditions.

### EEG Spectral Power

*Total* EEG spectral power to the very brief apparent motion transition generated consistent prolonged bursts of activity in theta, beta and gamma EEG frequency bands which overall behaved similarly across conditions in a task requiring detecting negatives of the stimuli (see Figure 4). We expected to observe oscillatory EEG changes in beta and gamma bands that would occur at different post-motion onset times for facial and control motion stimuli when statistical comparisons were made between conditions, in line with our previous study where participants detected color changes in line drawn face and control motion stimuli (Rossi et al., 2014). Statistically significant differences between stimulus conditions were confined to the beta and gamma bands only. The main significant change in gamma activity occurred at ~200 and ~300 ms post-motion onset for LINE faces bilaterally, but only in the left electrode cluster for REAL faces. Direct gaze transitions elicited stronger gamma amplitudes at ~200 ms (for LINE and REAL faces), whereas averted gaze elicited stronger gamma amplitudes at ~300 ms. We speculate that these bursts of activity reflect processing of facial information, as these changes were not present in the respective control conditions (compare first and second rows of Figure 5). REAL controls showed a gamma amplitude increment (~40–50 Hz) for the Direct condition, which occurred ~100 ms later than the gamma burst seen for REAL faces (compare second and last rows, Figure 5). It may be that this gamma burst to the controls might be a general coherent motion effect in data that were sampled from our occipito-temporal electrode clusters in a task where negative images of stimuli had to be detected.

In our previous study, we examined ERSP changes to impoverished line-drawn faces and controls only, where facial movements included eye and mouth movements in a color detection task that required a behavioral response for all presented stimuli (Rossi et al., 2014). In that study we also observed significant transient increases in the beta and gamma ranges to the facial motion stimuli, but these changes in oscillatory activity tended to occur at different time points relative to those seen in the current study. Gamma range changes to eye and mouth movements, if present, occurred much later in time relative to the current study e.g., after 400 ms post-motion onset relative to the changes at ~150–300 ms in the current study and favored direct gaze and mouth closing movements. Beta range changes, if present, showed a short burst at ~100 ms and a more prolonged burst between 350–550 ms favoring averted gaze (Rossi et al., 2014). In our previous study (Rossi et al., 2014) subjects viewed line-drawn face displays where the color of the lines could be either white or red, with subjects having to make a color decision on every seen motion transition. In our current study, as we had both line-drawn and real faces in the same experiment, we elected to use a target detection task where subjects had to identify negatives of all stimulus types, to try to ensure that equal attention was given to all stimulus types. Taken together, the ERSP findings of both studies would indicate that the significant differences in oscillatory behavior we observed at sites producing maximal ERP activity might be driven rather by task differences/decisions rather than the motion characteristics of the stimuli *per se*. Having said that, in both studies, changes in oscillatory activity were different across faces and respective controls, suggesting that changes in total oscillatory activity post-motion onset may reflect a complex mix of task and stimulus-related properties. At this point in time much more data are needed to make sense of these changes in oscillatory activity to facial motion stimuli and their relationship to ERP activity—it is not yet customary to perform both types of analysis in studies.

Caruana et al. (2014) performed an intracranial EEG study where ERPs and oscillatory activity were examined side-by-side. They presented epilepsy surgery patients with faces showing dynamic gaze changes while intracranial EEG was recorded from electrodes sited in the posterolateral temporal cortex. Intracranial N200 ERP activity (the analog of the scalp N170) and transient broadband high frequency gamma band activity (out to 500 Hz) occurring at around the same time as the N200 was significantly larger when patients viewed averted gaze relative to either direct gaze or lateral switching movements (Caruana et al., 2014). Lachaux et al. (2005) reported intracranial gamma-band activity in left fusiform gyrus, occipital gyrus and intraparietal sulcus for a static face detection task. Gamma band amplitude (40–200 Hz) significantly increased between 250–500 ms post-stimulus onset, and these condition differences were not present in the ERP (Lachaux et al., 2005). In contrast, intracranial N200 activity and high frequency gamma activity in ventral temporal cortex to viewing static faces has been documented (Engell and McCarthy, 2011). The presence of gamma activity in the ERSP predicted the presence and size of the N200 at a particular site. Relevant to this study, however, N200 activity was elicited to impoverished face stimuli, but notably gamma activity was absent (Engell and McCarthy, 2011), indicating that the relationship between intracranial ERP activity and gamma activity can be a very complex one. It is difficult to make a comparison between intracranial and scalp EEG studies, because with intracranial EEG high frequency gamma band activity can be sampled, whereas in scalp EEG studies the skull effectively acts as a low pass filter so that gamma frequencies typically will be recorded only under 100 Hz (Srinivasan et al., 1996). Interestingly, impoverished face stimuli, as used in Engell and McCarthy (2011) study and our study, do not appear to generate prominent gamma activity. In our study, stimuli tended to evoke sustained activity in frequency ranges below gamma), gamma activity was transient and was seen at around 200 ms post-motion onset in electrodes sited over lateral temporal scalp (Figure 4), similar to the broadband gamma reported in the intracranial EEG studies (e.g., Lachaux et al., 2005; Engell and McCarthy, 2011; Caruana et al., 2014). In another scalp EEG study, Zion-Golumbic and Bentin (2007), noted that activity in the 25–45 Hz range between 200–300 ms post-stimulus onset, was largest for (static) real faces compared to scrambled real faces in midline parieto-occipital locations (Zion-Golumbic and Bentin, 2007).

In our study, it is not entirely clear if the gamma activity could be related to configurality, movement, or a combination of both. However, we believe that the gamma responses at ~200 and ~300 ms present both for LINE and REAL stimuli reflect a preferential response to facial movement. An alternative explanation is that as a gamma response was seen for LINE and REAL faces and controls, albeit at different post-motion onsets, these gamma responses might be correlates of motion perception in the horizontal axis (see Figure 5). However, in our previous study we included mouth movements (with a large vertical component) in an impoverished face, and recorded gamma activity to both stimulus types (Rossi et al., 2014). From the relatively few existing studies in the literature, it is clear that the relationship between oscillatory EEG activity, including gamma, and stimulus and task type is complex. Much further work to viewing motion stimuli with different attributes (e.g., linear vs. non-linear, inward vs. outward radial, looming vs. receding) where ERP and ERSP activity are directly compared will be needed to disentangle these issues.

As noted earlier, LINE FACES and CONTROLS differ in configuration, but produce identical movements. A common spectral change between LINE FACES and CONTROLS was a decrement in beta power, maximal at ~25 Hz for the conditions producing direct gaze (FACES) and a diamond shape (CONTROLS), albeit at slightly different time intervals (FACES from ~215–300 ms, CONTROLS ~300–400 ms). Since the common feature between these stimulus types is apparent motion of identical numbers of pixels, these beta components might be related to the encoding of the movement. Beta spectral power has been previously associated with the perception and production of movement *per se* (Pfurtscheller et al., 1996; Müller et al., 2003; Müller-Putz et al., 2007). However, the beta change for LINE FACES occurred earlier than that for LINE CONTROLS. We have previously seen similar decreases in the 20–30 Hz beta range for line-drawn faces producing identical lateral eye movements as those used in the current study (Rossi et al., 2014). However, in Rossi et al. (2014) decrements in beta spectral power occurred later (~400–550 ms). In these two studies, participants were asked to respond to very different stimulus attributes, and with different stimulus probability. In Rossi et al. (2014) participants responded *on every trial* to indicate the color of the line-drawn stimulus (which varied randomly from white to red, and *vice-versa)*. In the current study, participants responded to infrequent targets that were negatives of all stimuli. Hence, at this stage it is not possible to clarify the nature of the observed beta power change to the apparent motion stimulus. We do believe that these differences were driven by task demands, and will have to explicitly test this in future studies.

## General Conclusions

We would advocate that future studies evaluate ERP *and* ERSP activity in parallel, so that we can develop an understanding of the functional significance of each type of neurophysiological activity, and how one might affect the other. Gaze changes produced gamma activity irrespective of face type: direct gaze elicited more gamma at an earlier latency relative to averted gaze. Overall, our N170 ERP peak analysis argues for the idea that gaze changes/eye movements in impoverished line-drawn faces do not trigger the neural responses that have been associated to the perception of socially relevant facial motion (relevant for communicative behavior), replicating data in an earlier study (Rossi et al., 2014). In contrast, real faces in this study as well as others (e.g., Puce et al., 2000, 2003; Conty et al., 2007; Latinus et al., revised and resubmitted) show N170 sensitivity to gaze changes. Interestingly, differences between our real faces and control stimuli were not as strong as we had previously demonstrated (Puce et al., 2000), and this may be due to ability to potentially visualize our current controls as faces. Overall our data indicate that N170s elicited to social attention manipulations are not modulated by top-down processes (such as priming or context) for impoverished faces.

Taking together the findings of our previous and current studies, N170s to gaze changes appear to be generated by different processes relative to mouth movements. Eye movements/gaze changes in real faces generate local visuospatial luminance/contrast changes producing an N170 that altered by the luminance/contrast change that occurs between changing gaze conditions. When gaze changes are presented in an impoverished face, there is no differential luminance/contrast change and the N170 does not show modulation across gaze conditions. In contrast, mouth opening and closing movements are a type of articulated biological motion, whose moving form modulates N170 irrespective of whether the movement occurs in a real or an impoverished face (Puce et al., 2003; Rossi et al., 2014), therefore largely independent of luminance/contrast. Yet, the motion of both face parts happens to elicit ERP activity at the same latency with a similar scalp topography that likely reflects an aggregate of neural activity from various parts of motion-sensitive, as shown clearly by fMRI studies (e.g., Puce and Perrett, 2003). The functional dynamics from this very heterogeneous brain network will likely only be disentangled by aggregating the data from a number of different investigations such as functional connectivity using fMRI, intracranial EEG and scalp EEG/MEG studies which examine evoked and induced neurophysiological activity in healthy subjects and individuals with neuropsychiatric disorders.

## Conflict of Interest Statement

The authors declare that the research was conducted in the absence of any commercial or financial relationships that could be construed as a potential conflict of interest. The Guest Associate Editor Davide Rivolta declares that, despite having collaborated with author Ania Puce, the review process was handled objectively and no conflict of interest exists.
